# Huge accessory spleen with torsion, mimicking splenic lymphangioma: A case report and review of the literature

**DOI:** 10.1016/j.ijscr.2023.108597

**Published:** 2023-07-29

**Authors:** Mujtaba Haidari, Haider Ali Malakzai, Ahmed Maseh Haidary, Fazel Rahman Faizi, Jamshid Abdul-Ghafar

**Affiliations:** a Department of Pathology and Clinical Laboratory, French Medical Institute for Mothers and Children (FMIC), Kabul, Afghanistan; b Human Medical Laboratories (HML), Kabul, Afghanistan; c Department of Radiology, Kabul University of Medical Science (KUMS), Kabul, Afghanistan

**Keywords:** Accessory spleen, Oversized, Torsion, Lymphangioma

## Abstract

**Introduction and importance:**

Approximately 30 % of population can have an accessory spleen, which is most often asymptomatic. Only when it becomes large in size, it may elicit symptoms, mostly due to complications, such as torsion, infarction, or traumatic hemorrhage. The preoperative diagnosis of an accessory spleen is often challenging due to its propensity to manifest the neoplasms of adjacent organs. Here we report a rare case of a huge tortured accessory spleen mimicking splenic lymphangioma and to review the literature.

**Case presentation:**

A 27-year-old man was admitted to the emergency department following left hypochondrial pain that lasted for three days. The computed tomography scan findings demonstrate the spleen in its normal position, showing smooth outlines attached to a similar-density cystic heterogenous mass exhibiting strong radiological evidence of splenic lymphangioma. Surgical excision was performed, and a large tortured accessory spleen was discovered that was attached to the lower pole of the spleen by connective tissue.

**Clinical discussion:**

An accessory spleen is always smaller than 4 cm, and accessory spleens larger than that, especially with torsion, are extremely uncommon. According to literature, the identification of accessory spleen on the basis of clinical and radiological features is very difficult, especially when the patient presents with symptoms of acute abdomen.

**Conclusion:**

Considering the fact that accessory splenic tissue can mimics neoplasms of the spleen or nearby organs, it should be included in the differential diagnosis in an undiagnosed pre- or intraoperative hypochondrial mass, located in the vicinity of the spleen.

## Highlights

• Accessory spleen can be found in 10 to 30 % of the papulation and has an asymptomatic nature.
• Preoperative diagnosis of the accessory spleen is very difficult because it usually mimics neoplasms of nearby affected organs.
• An accessory spleen is always smaller than 4 cm, and accessory spleens larger than that, especially with torsion, are extremely uncommon.
• Most of the accessory spleen are diagnosed by pathological examination and during surgery.

## 1 Introduction

Embryological origin of the spleen is from the overlying epithelium of the pancreatic endoderm in the sixth week of intrauterine life [[1], [2], [3]]. It is the largest lymphoid organ of the body, with an average weight of 200 g, located between the left hemidiaphragm and the greater curvature of the stomach [2]. Development of the spleen during embryonal life can be affected by various congenital factors that may give rise to various structural and anatomical abnormalities of the spleen, such as polysplenia, persistent lobulation, accessory spleens or even complete agenesis of the organ [2]. Accessory spleen (AS) also known as splenunculi or supernumerary spleen, is a term used to describe the congenital form of ectopic splenic tissue, usually located in splenic hilum that can be found in nearly 30 % of the general population [4]. Considering the size, such AS tissue is usually less than 4 cm in size [2,3]. As per our extensive literature review, there were only 7 reported cases of accessory spleen with size larger than 8 cm, as shown in Table 1. ASs are mostly asymptomatic and are found incidentally during radiological investigations for other unrelated pathologies [4]. When an AS becomes oversized, it may become symptomatic, usually with right upper quadrant discomfort, as well as symptoms related to complications such as torsion, necrosis, infarction, or traumatic bleeding [1,5]. Most of the reported articles in the literature emphasized on the difficulty of reaching a diagnosis before surgery, especially when there is emergency presentation, as in our patient, where the signs and symptoms can mimic neoplasms of affected or nearby organs [3]. In most of the reported cases, preoperative diagnosis of ASs was in favour of solid tumors [3,4,[6], [7], [8]]. Finally it has to be mentioned that only rare cases of AS with torsion or infarction have been reported, which can radiologically mimic mesenteric cysts or lymphangiomas [6,9,10]. In this report we presented probably the first case of a huge tortured AS with infarction, mimicking splenic lymphangioma on computed tomography scan evaluations. This study was reported in line with the SCARE Criteria [11].

**Table 1:** Reported cases of extra-pancreatic accessory spleen with size of 8 or larger than 8 cm.

Reported cases of extra-pancreatic accessory spleen with size of 8 or larger than 8 cm.							
Seq no.	Article (year) (reference)	Age (Y) & gender	Torsion	Clinical presentation & radiological diagnosis	Size of AS	Diagnosed by	Location and side of the accessory spleen
1	Pietro (2009) [16]	12/M	Yes	Abdominal pain with vomiting and diarrhea for a day.	8.5 cm	Histopathology	Anteromedial
2	Cheng Zhuan (2011) [24]	22/F	No	Dull pain at LUQ of abdomen with diarrhea for 3 months. Round mass with abundant blood supply and similar density to spleen.	12 cm	Surgery	Left side of greater omentum
3	Arra (2013) [12]	24/M	No	Right sided abdominal mass on self-examination. Radiologically suspected of malignant adrenal tumor.	20 cm	Surgery	Right side supra renal region
4	Vyacheslav (2014) [25]	20/F	Yes	5-day history of left-sided abdominal pain. Giant accessory spleen with ischemia.	12 cm	Radiology	Posteromedial
5	Maharaj (2016) [23]	44/M	No	Right upper quadrant pain with radiation to back. Radiologically suspected of duodenal GIST and retro-peritoneal sarcoma.	11 cm	Surgery	Right side of RPS
6	Yourong Feng (2018) [26]	39/F	No	Sudden lower abdominal pain for a week. Radiologically suspected of pelvic neoplasm or GIST.	8 cm in the largest one	Histopathology	Multiple pelvic round masses
7	Youjian Li (2019) [1]	31/F	No	14-year LUQ pain after running. Round well-defined mass at right renal hilum.	9 cm	Histopathology	Left side RPS
8	Current case (2023)	27/M	Yes	LUQ pain for 3 days with radiological suspicion of splenic lymphangioma	15 cm	Histopathology	LUQ

Abbreviations: AS; accessory spleen, LUQ; left upper quadrant, RPS; retro-peritoneal space, GIST; gastrointestinal stromal tumor.

## 2 Case presentation

**Fig. 1:**
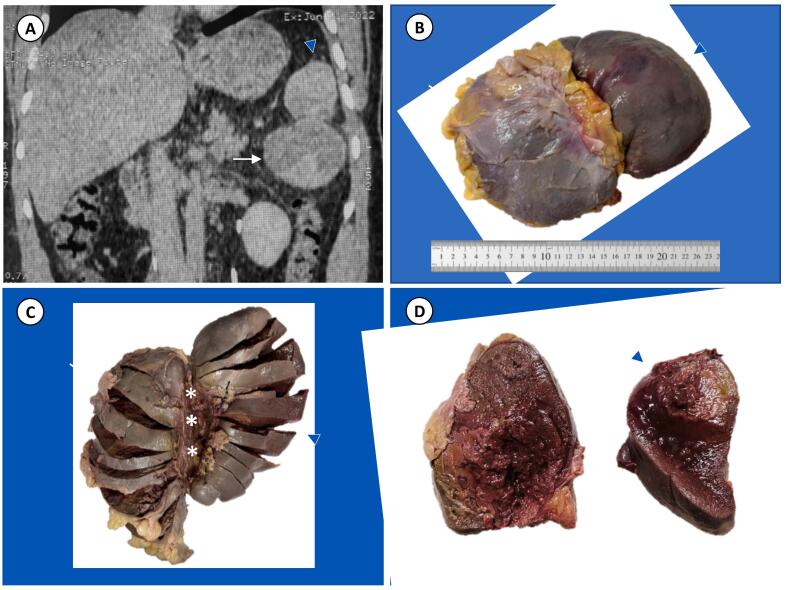
(Arrow; spleen, arrow head; accessory spleen). (A): Coronal reconstructed post-contrast CT images, showing the spleen in its normal position with homogeneous density and smooth outlines attached with another cystic heterogenous lesion with similar density at the lower pole of the spleen, compressing the left kidney. (B): Intact gross image of spleen attached to similar large mass. (C): The spleen and the AS are attached with each other by connective tissue (stars). (D): Cut surface of the spleen and accessory spleen exhibiting similar dark-red infarcted cut surface with persevered sub-capsular region. (For interpretation of the references to colour in this figure legend, the reader is referred to the web version of this article.)

**Fig. 2:**
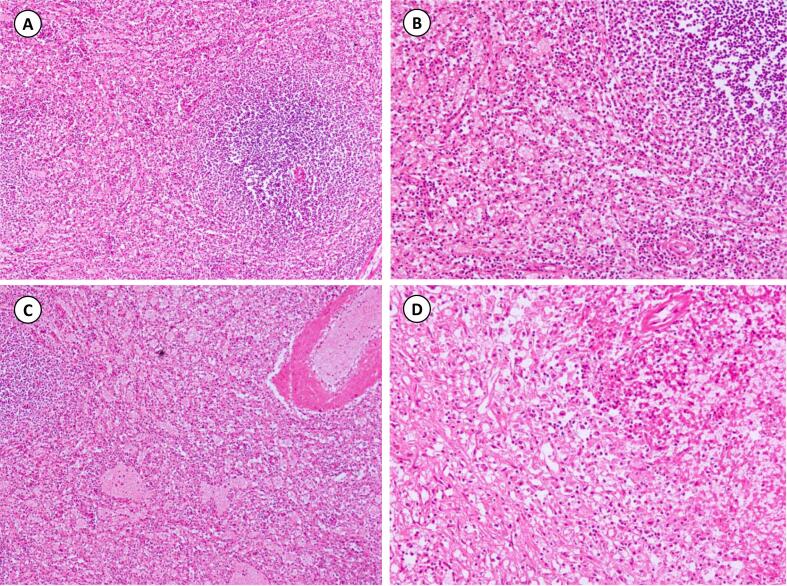
(A): Showing the microscopic image of spleen with infarcted changes, hemorrhage, red, and wight pulps (H&E, ×40). (B) The accessory spleen is also revealing splenic parenchyma exhibiting devitalized changes, hemorrhage, red, and wight pulps (H&E, ×60). (C & D): Exhibiting the microscopic findings of dilated blood vessels with infarcted areas (H&E, ×40 & ×60 respectively). (For interpretation of the references to colour in this figure legend, the reader is referred to the web version of this article.)

A 27-year-old man was admitted to the emergency department due to persistent left hypochondrial pain of three days duration. The patient was haemodynamically stable, with a blood pressure of 100/60 mmHg and a pulse rate of 88/min. Laboratory exams, including peripheral blood counts and tumor markers (CEA and CA125), were normal. The post-contrast CT scan from the abdomen demonstrates the spleen in its normal position, showing homogeneous density with smooth outlines accompanied by another cystic heterogenous lesion with similar density as that of the spleen in the left upper quadrant, that was attached to the lower pole of the spleen, compressing the left kidney, with no infiltration or invasion of surrounding structures, exhibiting radiological evidence strongly suggestive of splenic lymphangioma (Fig. 1A). An exploratory laparotomy commenced with a midline abdominal incision, followed by the resolution of the torsion. An accessory spleen, having texture as well as consistency similar to the spleen, was found to be attached to the actual spleen. It was gently grasped and displaced medially towards the incision. After the separation of avascular peritoneal attachments and ligaments by electrocautery, the short gastric vessels were identified and then ligated. Splenectomy with excision of the attached large mass was performed and sent for histopathological evaluation. Grossly, the specimen was in its intact form, comprising of a spleen and an attached similar mass, shown in Fig. 1B. The spleen marked with the black suture, measured 12 × 5 cm, exhibiting a smooth external surface and an intact capsule, which was attached to AS by connective tissue and measures 15 × 7 cm (Fig. 1C). On serial sectioning of the spleen and the mass, they reveal similar dark-red hemorrhagic infarcted cut surfaces (Fig. 1D). Microscopic findings from the spleen and the mass both revealed splenic parenchyma consisting of red and white pulp with diffuse hemorrhage, dilated blood vessels, and areas of infarction, as shown in Fig. 2 that lead to the diagnosis of an AS with torsion. After 1.5 years of follow up and a second CT scan, the patient was well with no complications.

## 3 Discussion

The spleen is one of the most important organs of immune system [12]. In fact, patients without a spleen, have high risk of infections by encapsulated bacteria, such as *Streptococcus pneumoniae*, *Hemophilus influenza* and *Neisseria meningitidis* [12]. Congenital abnormalities of the spleen include Asplenia, splenosis, hyposplenia, persistent lobulation, and ASs [2]. By definition, splenosis is an acquired alteration, and asplenia is the complete absence of the spleen [2]. On the other hand, an accessory spleen is defined as ectopic splenic tissue that develops due to the failure of cell fusion during migrating from the midline to the left upper quadrant [12,13]. Such ASs are usually located at the hilum and vascular pedicle of the actual spleen. However, cases have been reported to be located in the tail of the pancreas, left ovary, left testis, greater omentum, curvatures of the stomach, mesentery of the intestine, and pouch of Douglas, some of which have been listed in Table 1. ASs are usually single and can be found in almost 30 % of the general papulation [4,14,15]. Macroscopically, they are composed of a solid mass usually measuring less than 4 cm in diameter with the sizes larger than 8 cm being very rare [16,17]. An accessory spleen commonly has a well-defined fibrotic capsule that separates the surrounding normal tissue from its parenchyma [3]. They are diagnosed during radiological examinations for other diseases. When an AS becomes oversized, then it results in pain in the right upper quadrant or the affected area, mostly due to complications such as torsion, necrosis, infarction, or traumatic bleeding [1,5]. AS can also cause acute abdomen pain, which should be differentiate from other causes of acute abdomen in both genders [[18], [19], [20]]. Sometimes, when the actual spleen is removed either post traumatically or for any other medical indications, the ASs undergoes compensatory enlargement, sometimes reaching a size of about 5 cm in its longest diameter [21].

Nuclear scanning utilizing colloid labeled with Technetium 99 can makes the spleen's parenchyma visible and recognizable and therefore, it is the only radiological modality of choice for confirmation of the diagnosis but also for accurately locating the ASs [22]. Unfortunately, nuclear medicine is not available in most of the diagnostic centers, therefore, often only surgical excision and histopathological evaluations can confirm the diagnosis [23].

Based on reviewed articles in the literature we found seven cases of ASs larger than 8 cm and only one reported case larger than our case which is listed in Table 1. Of seven cases, only one case had an established perioperative diagnosis of AS. The differential diagnoses ranged from adrenal gland tumors, sarcomas, cystic lesions like hemangiomas. In all cases the correct diagnosis of AS was established either at surgery or after pathological examination.

## 4 Conclusion

Our extensive literature review demonstrated that the diagnosis of AS is difficult, especially when the presentation is as an acute abdomen or as is in our case. The misdiagnosis is mostly including neoplastic disease, lymphadenopathies, and cysts, which leading to unnecessary excision of the ASs. Therefore, an undiagnosed pre- or intraoperative mass, which is located closely to the spleen have to be managed carefully. Intra-operative evaluation and histopathological examination remain as the modalities of choice for accurate diagnosis of AS.

## Consent

Written informed consent was obtained from the patient for publication of this case report and any accompanying images. A copy of the written consent is available for review by the Editor-in-Chief of this journal.

## Ethical approval

Since this was a retrospective observational study and did not involve actual patient, patient's images or videos, it was granted an exemption from requiring ethics approval from the institutional Ethics Review Committee (ERC).

## Funding

No financial support was provided for this study.

## Guarantor

Jamshid Abdul-Ghafar, MD, PhD, FRCPath

## Research registration number

N/A.

## CRediT authorship contribution statement

JAG and MH diagnosed the case. MH conceived the idea. MH contributed to the writing of the manuscript's first draft. JAG, AMH and HAM were major contributor to the critically revising of the manuscript and important intellectual content. FRF was a major contributor of radiological image description. All authors read and approved the final manuscript.

## Declaration of competing interest

The authors declare that they have no competing interests.

## Data Availability

All data generated are included in this article.
